# Survival analysis of adult and children intermittent exotropia using a matched case-control design

**DOI:** 10.1038/s41598-018-38160-8

**Published:** 2019-01-24

**Authors:** Daye Diana Choi, Hoon Noh, Kyung-Ah Park, Sei Yeul Oh

**Affiliations:** Department of Ophthalmology, Samsung Medical Center, Sungkyunkwan University School of Medicine, Seoul, Korea

## Abstract

To compare the surgical outcomes of adult intermittent exotropia (X(T)) patients and matched control children X(T) patients including survival analysis. Fifty-two adult X(T) patients and 129 matched control children X(T) patients were included. Clinical characteristics, survival analysis, and surgical dose-response curves were evaluated and compared between the two groups. The weighted Cox proportional hazards regression analysis was used in order to find risk factors for the recurrence. Using Kaplan-Meier survival analysis, the cumulative probability of survival rate considering recurrence as event of Adult group were 93.97% for one year, and maintained at 88.44% for two, three. four, and five years after surgery. In contrast, those of the Child group were 83.6%, 76.5%, 65.6%, 56.23%, and 40.16% for one, two, three, four, and five years after surgery, respectively. The Adult group had a better event-free survival curve than the Child group as analyzed by a Log-rank test (p = 0.020). According to multivariate weighted Cox regression analysis, the younger age at operation and the larger preoperative angle were significant risk factors for recurrence.

## Introduction

Intermittent exotropia (X(T)) is one of the most common types of strabismus in Asia^1,2^. Because the symptomatic onset of X(T) is usually during early childhood, most of the studies on X(T) only include patients who are children. Only a few studies have discussed the clinical characteristics and surgical outcomes of adult X(T) patients, which were different from those of children X(T) patients’^3–5^. According to a review by Kushner and Mills, strabismus surgery for adults can achieve successful outcomes^6,7^. While controversial, there are reports that an older age at operation is associated with a lower recurrence rate^8^. Comparing between adult and children X(T) patients might also give us insight into the factors leading to a high recurrence rate in child X(T) surgery. Recurrence is the most common problem following X(T) surgery in children, causing many surgeons to intentionally prefer some degree of overcorrection^9,10^. In the present study, we compared the surgical outcomes of adult X(T) patients and matched control children X(T) patients in terms of survival analysis considering recurrence as failure. Weighted cox proportional regression analysis was also done in order to find the risk factors of recurrence for both groups, and a dose-response curve comparison was performed between the two groups.

## Material and Methods

### Patients

From January 2003 to June 2016, a total of 242 adult patients aged over 40 underwent strabismus surgery due to exotropia from our clinic. Of these, 190 patients were excluded from this study because of sensory (moderate to severe amblyopia), paralytic, or restrictive strabismus, a consecutive history of prior strabismus surgery, and insufficient follow-up, leaving a final of 52 adult subjects for analysis. Patients with A or V patterns, dissociated vertical deviation, or oblique muscle overactions not requiring surgery were included. In order to compare surgical outcomes between children and adult with similar distributions of some characteristics, we generated a matched child dataset using a propensity score matching method from the whole patient list who underwent strabismus surgery before age 19. To evaluate the propensity scores, we considered a multivariable logistic regression model for two groups with covariates: preoperative angle of deviation, sex, and the length of follow up time, and controlled the standardized mean difference (SMD) between the two groups to be less than 10% for those covariates. After matching, we had a 1:3 matched dataset, 129 child and 52 adult subjects.

### Preoperative ophthalmologic examination

The following preoperative characteristics were collected from medical records: sex, age at operation, the length of follow up, type and magnitude of strabismus surgery, angle of deviation at distance and near, type of exotropia, presence of lateral incomitance, spherical equivalent, best corrected visual acuity (BCVA), presence of amblyopia, anisometropia, dissociated vertical deviation, vertical deviation, superior oblique or inferior oblique overaction, and office control scale. Prism and alternate cover testing was performed at 6 m and 33 cm. An additional near prism and alternate cover test was performed after 1 h of monocular occlusion for any patients with the smaller near deviation than the distance deviation. Divergence excess type was defined as >10PD larger exodeviation at distance than at near. Lateral incomitance was defined as >5 PD change in right or left gaze from the primary position. Refractive error was determined using manifest refraction for adult patients and cycloplegic refraction for child patients as spherical equivalent values. To exclude sensory exotropia, patients with BCVAs lower than 20/100 in the worse eye were excluded in this study. Amblyopia was defined as a difference of two lines or more in BCVA. Anisometropia was defined as a spherical equivalent difference of >3.0 D between the two eyes. The office control scale was divided into three ratings (Good, Fair, Poor). All preoperative ophthalmologic examinations were conducted by a single clinician (S.Y.O.).

### Intraoperative procedure

All operations were performed by a single surgeon (S.Y.O). Patients with an exodeviation under 20 PD underwent unilateral lateral recession (ULR), and the rest underwent bilateral lateral rectus recession (BLR) or lateral rectus recession and medial rectus resection (RR). Surgical dose was based on the largest preoperative exodeviation at distance or near. The surgical table is presented in Table 1. In the cases of muscle adjustments, the procedure was performed under topical anesthesia two to three hours postoperatively when the patients were alert enough to cooperate with orthoptic measurement. The aim of adjustment was to make orthophoria without diplopia.

**Table 1:** Surgical tables of unilateral and bilateral lateral rectus muscle recession and lateral rectus muscle recession and medial rectus resection in intermittent exotropia.

Preoperative	ULR (mm)	BLR (mm)	RR (mm)	
Deviation (PD)			LR recession	MR resection
15	9.0			
20	9.5			
25		6.0 × 2	6.0	4.5
30		7.0 × 2	6.5	5.0
35		7.5 × 2	7.0	5.5
40		8.0 × 2	7.5	6.0
50		9.0 × 2	8.0	6.5

ULR = unilateral lateral rectus recession, BLR = bilateral lateral rectus recession, RR = lateral rectus recession and medial rectus resection, PD = prism diopters.

### Postoperative measurement

Postoperative alignment at distance and near deviation were measured at the first week, one, six, and 12 months after, and every year after surgery. Success was determined when the ocular deviation in the primary position was between <10 PD of exotropia and ≤5 PD of esotropia at far and near distance. Recurrence was defined as exodeviation of 10 PD or more at any time after the operation at far or near distance. Overcorrection was defined as esodeviation of 5 PD or more, but anyone with constant diplopia due to esotropia (even smaller than <5PD) or prescribed base out prism glasses were also defined as showing overcorrection.

### Statistical analysis

Statistical analyses were performed using SAS Enterprise Guide, version 9.4 (SAS Institute Inc, Cary, NC). The weighted t test, weighted x2 test, and Fisher exact test were used in order to compare the patients’ characteristics and their surgical outcomes. Weighted Cox proportional hazards regression analysis was used to find risk factors associated with recurrence after operation. Multi-colinearity using variance inflation factors was also considered. The cumulative probabilities of success were assessed according to the Kaplan-Meier life-table analysis. A log-rank test was used to compare the survival rates between the Adult and Child groups. The surgical dose-response curves were analyzed using linear regression analysis. *P* values less than 0.05 were considered to be statistically significant.

This study was performed in accordance with the tenets of the Declaration of Helsinki. Approval to conduct this study was obtained from the Institutional Review Board of Samsung Medical Center. Informed consent was waived by Institutional Review Board of Samsung Medical Center, because this study was conducted retrospectively using medical records without identifiable private information. Even though the surgical operation was conducted upon the subjects of this study, all procedures were planned and performed according to usual clinical care regardless of this study, thus there was no risk to the subjects.

## Results

Among 52 adult patients, 43 patients (82.69%) achieved successful outcomes, four patients (7.69%) experienced recurrence during follow up, and five patients (9.62%) remained overcorrected until the last follow-up. Out of five overcorrected adult patients, three had esotropia more than 5 PD, but the remaining two had only 2PD and 4PD esotropia with constant diplopia also classified as overcorrection according to our definition. We included 129 children patients as the matched control Child group, and 91 patients (70.54%) achieved successful outcome, 36 (27.90%) had recurrence during follow up, and two (1.55%) patients remained overcorrected until the last follow-up. At postoperative 1 week, 7 patients (13.46%) in adult group and 13 patients (10.07%) in children group showed larger than 2PD of esotropia at distance or near. The mean angle of deviation at postoperative 1 week was 0.86PD exotropia at distance and 1.60PD exotropia at near in Adult group and 0.40PD exotropia at distance and 0.68PD exotropia at near in Child group. The average postoperative follow-ups were 71.9 months in the Adult group and 79.6 months in the Child group.

The baseline characteristics of the Adult and Child group are shown in Table 2. The Adult group was more myopic and had more anisometropic patients than the Child group. The adult group also had more superior oblique over-action than the Child group. Unilateral LR recession and MR resection was the most common surgical choice for the Adult group, while bilateral LR recession was most common in the Child group. Because of the issue of cooperation, over 78% of the Adult group underwent adjustable suture, but only two (1.6%) patients underwent adjustable suture in the Child group. Lastly, the grade of office control was better in the Adult group than in the Child group.

**Table 2:** Preoperative characteristics of child and adult intermittent exotropia subjects.

	Adult group (N = 52)	Child group (N = 129)	p-value
Sex (Male: Female)	20: 32	53: 76	1.000^a^
Age at operation (year)	53.0 ± 9.46	5.6 ± 3.18	<**0.0001**^**b**^
Follow up time (months)	71.96 ± 79.75	79.59 ± 44.71	0.518^b^
Best-corrected visual acuity			
OD (logMAR)	0.09 ± 0.17	0.06 ± 0.10	0.813^b^
OS (logMAR)	0.09 ± 0.17	0.06 ± 0.09	0.765^b^
Spherical equivalent			
OD (diopters)	−1.60 ± 2.74	−0.15 ± 1.02	**0.015** ^**b**^
OS (diopters)	−1.78 ± 3.25	−0.27 ± 0.99	**0.005** ^**b**^
Preoperative deviation			
Distance (PD)	34.62 ± 13.94	31.68 ± 10.50	0.928^b^
Near (PD)	34.91 ± 14.49	32.16 ± 10.89	0.791^b^
Associated features			
Vertical deviation (PD)	2.93 ± 7.83	0.72 ± 2.88	0.063^b^
Dissociated vertical deviation	4 (7.7%)	4 (3.1%)	0.280^b^
Superior oblique overaction	7 (13.5%)	3 (2.3%)	**0.003** ^b^
Inferior oblique overaction	4 (7.7%)	19 (14.8%)	0.162^a^
Lateral incomitance	3 (5.8%)	3 (2.33%)	0.356^c^
Type of exodeviation			
Basic	50 (96.2%)	120 (93.0%)	0.379^b^
Divergence excess	2 (3.8%)	9 (7.0%)	
Operation type			**0.031** ^**b**^
Unilateral LR recession	12 (23.1%)	22 (17.1%)	
Bilateral LR recession	18 (34.6%)	80 (62.0%)	
LR recession & MR resection	22 (42.3%)	27 (20.9%)	
Adjustable suture	41 (78.8%)	2 (1.6%)	<**0.0001**^a^
Amblyopia	6 (11.5%)	9 (7.0%)	0.323^b^
Anisometropia	3 (5.8%)	1 (0.8%)	<**0.023**^**b**^
Office control			
Good: Fair: Poor	11: 7: 8	5: 55: 19	**0.049** ^**d**^

^a^weighted x^2^ test, ^b^weighted independent t test, ^c^weighted Fisher exact test, ^d^linear by linear test, PD = prism diopter, LR = lateral rectus, MR = medial rectus, SE = spherical equivalent.

**Figure 1:**
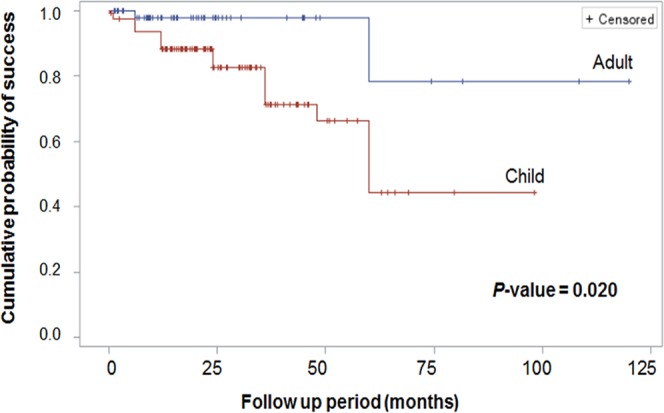
Kaplan-Meier survival analysis showing the recurrence-free survival curve after operation. Event-free survival curve is different between the adult and children groups (*p* = 0.020). *p*-value was calculated by log-Rank test. Survival rate of Adult group was 93.97% for one year, and maintained at 88.44% for two, three, four, and five years after surgery. In contrary, the survival rates of the Children group were 83.6%, 76.5%, 65.6%, 56.23%, and 40.16% for one, two, three, four, and five years after surgery, respectively.

Using Kaplan-Meier survival analysis, the cumulative probability of success rate considering recurrence as the event of the Adult group was 93.97% for one year, and maintained at 88.44% for two, three, four, and five years after surgery. In contrast, that of the Child group was 83.6%, 76.5%, 65.6%, 56.23%, and 40.16% for one, two, three, four, and five years after surgery, respectively. The Adult group had a better event-free survival curve than the Children group as analyzed by Log-rank test (*p* = 0.020, Fig. 1)

We performed weighted cox proportional hazards regression analysis in order to find the risk factors of recurrence in the Adult and Child groups. In multivariate analysis, a younger age at operation (HR = 0.979, CI = 0.960–0.998, *p* = 0.033), larger preoperative angle (HR = 1.046, CI = 1.011–1.081, *p* = 0.009) and divergence excess type (HR = 2.808, CI = 1.007–7.833, *p*-value = 0.049) were significant risk factors for recurrence (Table 3).

**Table 3:** Weighted Cox proportional-hazard regression analysis of recurrence in matched dataset (129 children and 52 adult subjects).

Variables	Univariate				Multivariate			
	p-value	Hazard ratio	95% CI lower	95% CI upper	p-value	Hazard Ratio	95% CI lower	95% CIupper
Sex (Male vs Female)	0.382	0.767	0.422	1.391				
Age at Operation	**0.019**	0.977	0.957	0.996	**0.033**	0.979	0.960	0.998
BCVA								
OD (logMAR)	0.083	0.347	0.104	1.150				
OS (logMAR)	0.377	0.555	0.151	2.048				
SE								
OD (diopters)	0.1765	1.196	0.923	1.550				
OS (diopters)	0.1045	1.303	0.947	1.793				
Operation type								
Overall	0.1893							
R&R vs BLR	1.000	1.113	0.545	2.272				
ULR vs BLR	0.1914	0.375	0.100	1.403				
Adjustable suture (Yes vs No)	0.0731	0.390	0.139	1.092				
Preop. deviation								
Distance	**0.001**	1.038	1.015	1.061	**0.009**	1.046	1.011	1.081
Near	**<0.001**	1.053	1.030	1.076				
Vertical deviation	0.931	1.003	0.941	1.069				
DVD (Yes vs No)	0.109	2.297	0.831	6.347				
SOOA (Yes vs No)	0.950	1.046	0.259	4.231				
IOOA (Yes vs No)	0.118	1.745	0.868	3.506				
Type of exodeviation (Divergence excess vs Basic)	**0.047**	2.721	1.015	7.297	**0.049**	2.808	1.007	7.833
Amblyopia (Yes vs No)	0.894	0.930	0.317	2.726				
Office control								
Overall	0.097							
Fair vs Good	0.8595	0.613	0.153	2.461				
Poor vs Good	0.8360	1.640	0.417	6.443				

BCVA = Best corrected visual acuity, SE = Spherical equivalent, R&R = unilateral lateral rectus recession and medial rectus resection, BLR = bilateral lateral rectus recession, Preop = preoperative, DVD = dissociated vertical deviation, SOOA = superior oblique muscle over-action, IOOA = inferior oblique muscle over-action.

**Figure 2:**
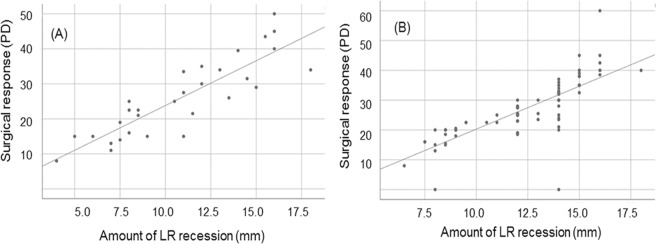
Dose-response curves of adult and child intermittent exotropia subjects who underwent lateral rectus recession. Scatter plot shows the surgical dose-response of (**A**) adult group and (**B**) child group using linear regression analysis based on alignment postoperative 1 week. There were no significant differences between the dose-response curves of the Adult and Child groups based on alignment 1 week postoperatively for lateral rectus recession (*p* = 0.486).

The surgical dose-response curves using linear regression analysis based on alignment one week postoperatively are shown in Fig. 2. The linear regression equation of the Child group who underwent LR recession was: change in deviation = 2.74599 + 2.74599 * amount of recession. The linear regression equation of Adult group who underwent LR recession was: change in deviation = 0.27878 + 2.55542 * amount of recession. The dose-response curve did not significantly differ between the two groups (*p* = 0.486 for LR recession, *p*-value by Log rank test, Fig. 2).

## Discussion

In our study, the Kaplan-Meier survival analysis of the Adult group had a significantly better event-free survival curve than the Child group. However, the dose-response curves were not significantly different between the Adult and Child groups, which implies the survival difference might be not due to the dose-response relationship. Even though we matched the preoperative deviation angle, sex, and the length of follow-up time, the refractive errors, simultaneous vertical strabismus, and the type of surgery were still different between the two groups. The refractive errors, simultaneous vertical strabismus, and the type of surgery all may influence the surgical outcomes in X(T)^11–14^. However, in multivariate weighted cox proportional hazard regression analysis of the Adult and Child groups, none of these factors was a significant risk factor for recurrence. Not conducting the adjustable suture and poorer office control were marginally associated with recurrence in univariate weighted cox proportional analysis, but these two factors were not included in the multivariate analysis. Instead, a larger preoperative angle, the divergence excess type, and a younger age at operation were the significant risk factors for recurrence in the multivariate analysis.

Some previous studies have concluded that there was no correlation between the degree of preoperative angle and recurrence rate^8,9^. However, other studies have found that a larger preoperative angle was associated with recurrence^14–16^. Our results also showed that the larger preoperative angle is a significant factor for recurrence (HR = 1.046, CI = 1.011–1.081, *p* = 0.009).

Although the divergence excess type was a statistically significant factor for recurrence in our study (HR = 2.808, CI = 1.007–7.833, *p*-value = 0.049), precaution is needed to interpret this result. The percentage of divergence excess type was very low overall (2 cases (3.8%) for Adult group and 9 cases (7%) for Child group), and three of them were classified as recurrence group. Considering the marginal significance of *p*-value and the small number of cases, the relationship between divergence excess type and the recurrence is not clear enough to discuss in the present study. Further studies focused on the relationship between the type of X(T) and the surgical outcome would be needed.

Whether the adjustable suture affects the surgical success rate in X(T) has been shown to vary. Mireskandari *et al*. concluded that the adjustable suture is indeed associated with a higher surgical success rate in primary surgery in adults with exotropia^17^. However, Bishop and Doran^18^ found no difference between the adjustable suture group and the non-adjustable suture group in their study. In the present study, the adjustable suture by itself failed to reach statistical significance in terms of affecting the surgical success considering recurrence as failure. However, this weak relationship might be explained by our policy of using the adjustable suture that is performed in a way to prevent overcorrection and postoperative diplopia, not to prevent future recurrence.

The relationship between the degree of the control grade and surgical success has been previously studied, and most of these studies have concluded that the control grade itself is not associated with surgical outcome^19,20^. However, some studies have stated that the control grade and stereoacuity are correlated, and that the degree of stereoacuity and surgical success are related^21,22^. In our result, the control grade was better in the Adult group than in the Child group, but the control grade was not a statistically significant risk factor for recurrence. Because our study did not include stereoacuity as a risk factor due to incomplete data, we are not able to discuss the relationship between the control grade, stereoacuity, and surgical outcome herein.

In the present study, younger age at operation was a risk factor for the recurrence in the X(T) (HR = 0.979, CI = 0.960–0.998, *p* = 0.033). Previous results have varied in concluding whether the age at the time of surgery is a determining factor for the success of the surgery. Abroms *et al*.^23^, Pratt-Johnson *et al*.^24^, and W.L. Asjes-Tydeman *et al*.^25^ published data showing that early surgery is associated with better surgical outcomes. Knapp also suggested early surgery for young patients so as to prevent sensory abnormalities and to minimized the tendency toward recurrence when the deviation angle is large^26^. On the other hand, SH Lim *et al*. analyzed the medical records of 489 subjects who received unilateral LR recession and MR resection procedure at a mean age of eight years, and found that an older age at surgery is associated with a lower recurrence rate^8^. However, Chia *et al*.^27^, Wilshaw *et al*.^28^, Beneish *et al*.^29^, Richard *et al*.^30^, Maruo *et al*.^31^, and Jeon *et al*.^32^ concluded that the age at operation did not matter in terms of surgical success. In spite of various results, these previous study results were not enough to evaluate the effect of age at operation, because these studies mostly included children X(T) patients only.

To the best of our knowledge, our study is the first comparing the surgical outcomes including survival analysis in Adult and Children X(T) patients. Previous studies have tried to find some age point for better surgical outcomes in the range of children, but were unclear. In this study, the survival curve was better in the Adult group than Child group. There are some possible reasons for this better survival outcome of Adult X(T) patients. First, the preoperative angle of Adult group might clearly be more accurate than Child group, because adult patients can cooperate much better than children patients. A more accurate preoperative deviation angle will lower the chance of undercorrection and overcorrection. Second, the Adult group had better office control than the Child group did preoperatively. Good office control might explain why the Adult group could delay the surgery till adulthood, and that also might be associated with good postoperative surgical outcomes, as discussed above^21,22^. Third, about 78% of the Adult group were able to undergo adjustable suture, while only 1.6% of the Child group underwent adjustable suture. Even though the adjustable suture was not a statistically significant factor in univariate weighted Cox proportional-hazard analysis (HR = 0.39, CI = 0.139–1.092, *p*-value = 0.073), we could guess that there is some marginal association between the adjustable suture and the surgical success rate^17^. Fourthly, the X(T) itself has a trend toward aggravation, which means the angle of deviation of exotropia becomes larger over time^33,34^. We matched the Child group to have the same amount of preoperative angle of deviation as the Adult group, however, according to the exodrift nature of X(T), the Child group might have a larger angle of exotropia when they reach the age of the Adult group without surgery. As the larger preoperative angle was the significant factor for recurrence in our study, the Child group might have worse prognosis in the future. Lastly, even though the pathophysiologic cause of X(T) is not clear, we can presume that the growth itself, including eyeball growth and brain maturation, might influence the course of X(T), and contribute to the variable surgical outcome. The more stable status of adults in terms of growth and brain maturation might be the reason for the better surgical outcomes of the Adult group.

Many surgeons prefer some degree of overcorrection after the surgery of X(T) in children patients, because of the high recurrence rate. However, our results showed that the Adult group not only had a low recurrence rate, but also had a low threshold to consecutive esotropia. Because we performed adjustable suture in over 78% of patients aimed at orthophoria and preventing overcorrection, only three adult patients out of 52 had esotropia larger than 5 PD. Two adult patients needed prism glasses because of diplopia even with only 2 PD and 4 PD postoperative esotropia. Thus, it is always safer to perform adjustable suture for adult X(T) patients to prevent overcorrection, and there is no need to overcorrect intentionally because of the low recurrence rate. Unlike the adult group, the cumulative survival rate of Child group showed an increasing recurrence rate over time. Lim *et al*. reported a cumulative probability of success rate of 22.1% at 5 years for children X(T) with a mean age of eight years^8^. Oh and Hwang also reported the cumulative probability of success at five years as about 45%^9^. Considering the increasing recurrence rate over time, it is clear that the longer follow up period is better for children X(T) patients.

This study has several limitations. First, this study is a retrospective case-control design, and even though we carefully selected the control Child group with several statistical considerations, the possibility of selection bias still exists and the results need careful interpretation. Second, the use of only a single institution might have limited the power of this study’s conclusion. However, comparison between the results of the two groups from a single surgeon could also have been a benefit in terms of the same amount of surgical table and identical surgical skills. Third, this study did not include sensory test results such as stereopsis and suppression, because many of the Child group were not able to perform the tests due to the cooperation problem.

In conclusion, this study compared the survival graphs considering recurrence as failure between Adult and Child X(T) groups, and the Adult group showed a better survival curve than the Child group. In multivariate weighted cox regression analysis, the younger age at operation and the larger preoperative angle were significant factors for recurrence in the Adult and Child groups. The surgical dose-response curves showed no significant differences between the Adult and Child groups.

## Data Availability

The datasets generated and analysed during the current study are available from the corresponding author on reasonable request.
